# Clinical and Translational Advances in Glioma Immunotherapy

**DOI:** 10.1007/s13311-022-01313-9

**Published:** 2022-10-27

**Authors:** Lukas Bunse, Theresa Bunse, Christopher Krämer, Yu-Chan Chih, Michael Platten

**Affiliations:** 1 DKTK Clinical Cooperation Unit (CCU) Neuroimmunology and Brain Tumor Immunology, German Cancer Research Center (DKFZ), Heidelberg, Germany; 2 Department of Neurology, Medical Faculty Mannheim, MCTN, Heidelberg University, Mannheim, Germany; 3 Faculty of Biosciences, Heidelberg University, Heidelberg, Germany; 4 Immune Monitoring Unit, National Center for Tumor Diseases (NCT), Heidelberg, Germany; 5 Helmholtz Institute of Translational Oncology (HI-TRON), Mainz, Germany; 6 DKFZ Hector Cancer Institute at the University Medical Center Mannheim, Mannheim, Germany

**Keywords:** Peptide vaccines, Personalized immunotherapy, Glioma immunotherapy, Combinatorial immunotherapy, T cell therapy, Neoepitopes, Glioma antigens

## Abstract

Gliomas are highly treatment refractory against immune checkpoint blockade, an immunotherapeutic modality that revolutionized therapy for many tumors. At the same time, technological innovation has dramatically accelerated the development of immunotherapeutic approaches such as personalized tumor-specific vaccine production, dendritic cell vaccine manufacture, patient-individual target selection and chimeric antigen receptor, and T cell receptor T cell manufacture. Here we review recent clinical and translational advances in glioma immunotherapy with a focus on targets and their cognate immune receptor derivates as well as concepts to improve intratumoral T cell effector functions.

**Supplementary Information:**

The online version contains supplementary material available at 10.1007/s13311-022-01313-9.

## Introduction

Standard of care (SOC) for glioma, one of the most malignant tumor entities and the most frequent primary brain tumor in the adult, is still confined to resection and radiochemotherapy, although preclinical research has put tremendous efforts into improvement of therapeutic approaches. These mainly include targeted therapy using small molecule inhibitors and immunotherapy. While conventional immunotherapies such as immune checkpoint inhibitors (ICI) are SOC in other entities such as melanoma, they mainly fail to confer clinical benefit for glioma patients. The reasons are manifold: rarity of well-defined specific antigens, impaired T cell recruitment, and a unique immunosuppressive and hypoxic microenvironment that differs from peripheral solid tumors, making glioma immunologically cold tumors. None of the phase 3 clinical trials using ICI in gliomas met their primary clinical endpoints for patients with newly diagnosed or relapsed glioblastoma (GBM) (CheckMate 143, CheckMate 498, CheckMate 548) [1, 2]. At the same time, chimeric antigen receptor (CAR) therapies have shown remarkable responses in some patients. With overall limited availability of GBM-specific extracellular antigens for CAR T cell therapy, vaccines targeting intracellular neoepitopes and tumor-associated antigens have demonstrated encouraging safety and feasibility results in phase 1 trials. Here, we summarize the current advances and further developments in antigen-targeted immunotherapies, including vaccines and adoptive T cell therapies, for glioma patients, with a focus on target selection, comparing self-antigens, neoantigens, and personalized approaches. ICI and oncolytic virotherapy as well as treatments targeting the immune microenvironment in glioma have been extensively reviewed elsewhere [3–6].

## Vaccine-Based Therapies for Malignant Glioma

### Conceptual Considerations on Vaccine Targets

Anti-cancer vaccines represent one — and, historically, the first — pillar of cancer immunotherapy. Vaccines have the advantages of a cost-effective and fast production, especially peptide vaccines, limited side effects, and the possibility to be combined to target multiple antigens. Yet, the stringency of suitable tumor-specific antigens has long impeded their clinical efficacy. For glioma, many glioma-associated targets, which are overexpressed in tumor cells compared to healthy tissue, or the so-called cancer-testis antigens, which are expressed in the germline, yet are invisible to the immune system due to lack of major histocompatibility complex (MHC) expression in this tissue, have been exploited as antigens for vaccination approaches. In 2000, Sahin et al. first described a comprehensive analysis of cancer-testis antigen expression in human glioma samples and concluded they may be exploitable for specific immunotherapy [7]. However, immune responses to such antigens are often prone to be suppressed by central tolerance towards these self-antigens, rendering such vaccines poorly effective, while on the other hand, if sufficient responses can be induced, autoimmune reactions via on-target side effects may occur, which negatively affects the safety of such vaccines. These considerations and the discovery that response to ICI positively correlates with tumor mutational burden (TMB) across cancer entities have provided a mechanistic basis for targeting mutated antigens, the so-called neoantigens, by vaccines due to their tumor-specificity [8, 9]. As most neoantigens generated by non-synonymous mutations are private, personalized approaches have been developed and translated into phase 1 clinical trials also in gliomas [10–12]. In the past, these have mainly focused on the induction of cytotoxic CD8+ T cell responses against neoepitopes presented on MHC class I. The majority of neoantigens, however, are presented on MHC class II molecules, stimulating CD4+ T-helper cell responses [13, 14]. The relevance of T-helper cells for anti-tumor immunity has long been neglected due to the scarcity of cytotoxic function; however, more recent observations underline their importance for cancer immunotherapy. For instance, numerous studies have demonstrated that T cell help by CD4+ T cells is required for anti-tumor efficacy of cytotoxic T cells [15–18]. Beyond this function, we and others have shown that strictly tumor antigen-specific CD4+ T cells are capable of and required for anti-tumor responses when induced by vaccination [14, 19]. In experimental sarcoma and glioma models, ICI efficacy depends not only on CD8+ , but also on CD4+ T cells [20, 21], and it has been shown that this also requires activity of antigen-specific CD4+ T cells in tumors with expression of MHC class II-restricted neoantigens [22]. Of note, since MHC class II-mediated antigen presentation is mainly achieved by professional antigen presenting myeloid cells such as dendritic cells (DCs) and macrophages, but also B cells, CD4+ T cell-mediated immune responses do not require MHC class II expression on tumor cells. These preclinical findings translate to clinical observations, demonstrating that while personalized neoepitope-specific vaccines induce CD4+ rather than CD8+ T cell responses in cancer patients [23, 24], mutation-specific CD4+ T-helper cells are capable of eradicating large tumors in cancer patients [25]. Efficacy of neoepitope-specific vaccines is not only determined by the number and antigenicity of the neoepitopes but also by their clonality and clonal representation within the tumor. Again, from immunotherapy using ICI, one can learn that neoantigen clonality is a key predictor for response, while subclonal neoantigens are prone to immune evasion due to negative clonal selection [26, 27]. Hence, driver mutations represent an attractive target for specific immunotherapies, as neoantigens derived from them are mostly clonal. Furthermore, such driver mutations are likely to be recurrent, hence shared between patient tumors, providing an opportunity for development of off-the-shelf vaccines. Pre-designed off-the-shelf vaccines do not require elaborate patient identification, regulatory processes, and manufacturing and are therefore more cost-effective than personalized neoantigen vaccines, and can usually be administered in a shorter time window [28].

### Vaccine Modalities

#### Peptide Vaccines

**Fig. 1:**
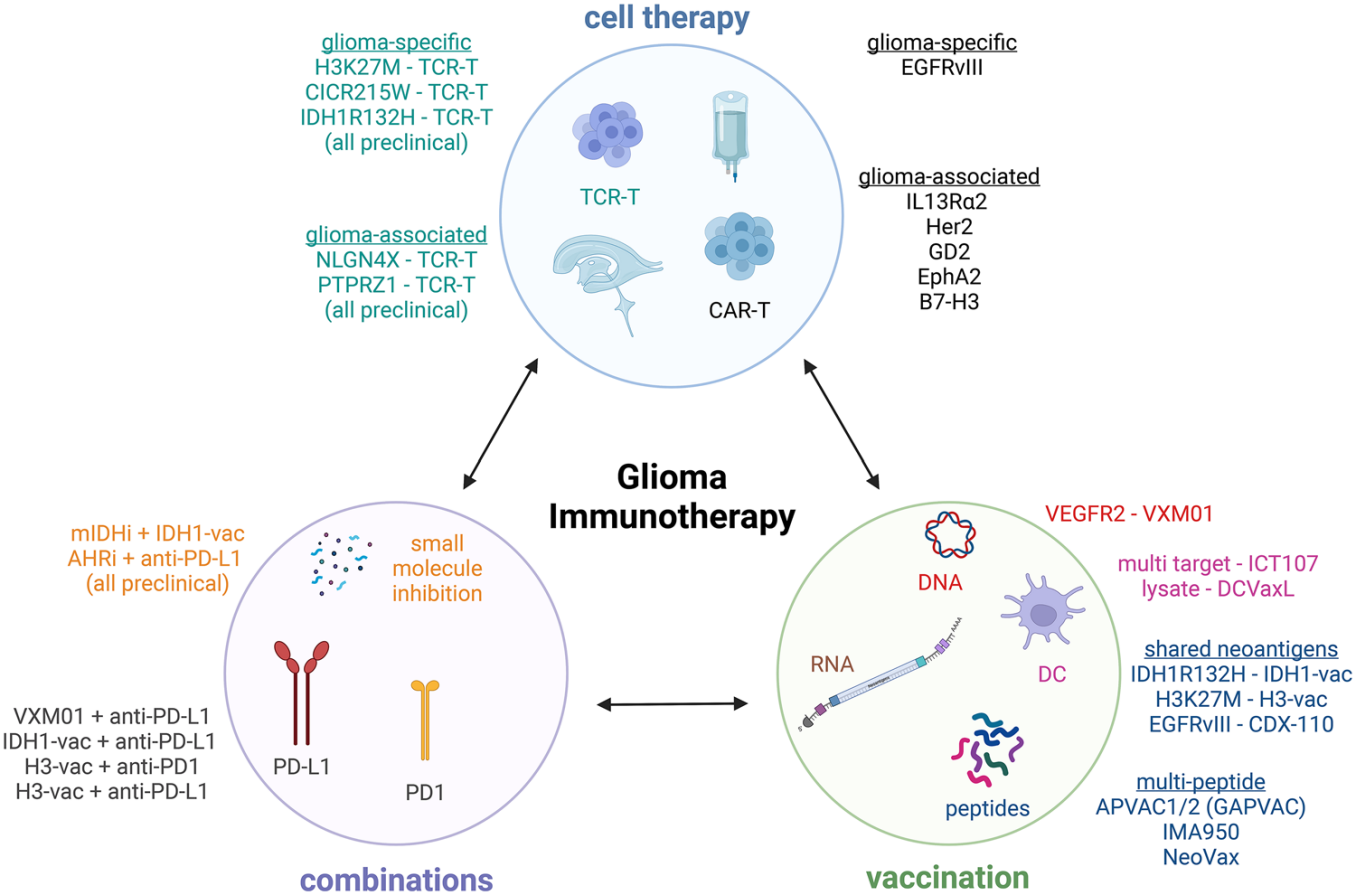
Modalities, targets, and combinatorial treatments in glioma immunotherapy and their interdependence

Peptide vaccines (Fig. 1) represent synthetic segments of protein antigens of various amino acid lengths, ranging from small MHC class I epitopes of 8–9 amino acids to long peptides of up to 50 amino acids, encompassing multiple MHC class I and II epitopes. While short peptides may be directly and exogenously loaded on MHC molecules, especially longer peptides are endocytosed by antigen-presenting cells, processed via proteolytic enzymes in the endosomal pathway or the proteasome before they are presented. Peptide vaccines require the concomitant application of adjuvants in order to stimulate antigen-presenting cells for efficient antigen presentation and co-stimulation of T cells. These can be toll-like receptor (TLR) agonists like imiquimod, granulocyte macrophage colony stimulating factor (GM-CSF), or synthetic double-stranded RNA molecules (poly-IC:LC). We will elaborate on peptide vaccines for glioma patients in detail below.

#### RNA Vaccines

In contrast to peptide vaccines, RNA vaccines represent potent adjuvants in themselves as free RNA is a TLR ligand. Improvements in formulation for stabilization, such as encapsulation in liposomes, allow simple intravenous injection of such vaccines and stable uptake by dendritic cells body-wide in lymphoid organs incl. spleen — which is the main compartment for antigen-presenting cells (APC) — lymph nodes, and bone marrow [29]. Vaccination with such RNA vaccines results in efficacious T cell priming, and therapeutic efficacy in various mouse tumor models. In the first phase I dose-escalation study, such liposomal RNA vaccine targeting four tumor-associated antigens is currently being tested for safety and efficacy in patients with advanced melanoma (NCT02410733). An exploratory interim analysis demonstrated vaccine-induced T cell responses in the vast majority of patients, which expanded over time as continuous vaccination was applied, and persisted to some extend in all patients due to a memory phenotype [30]. Some clinical responses as well as synergism with anti-programmed cell death protein 1 (PD1) checkpoint inhibitor therapy were detected in treated patients, who were ICI-experienced patients. Full trial data and subsequent clinical studies are required to draw final conclusions on the therapeutic efficacy and potential combinations for these vaccines. As of now, they have not been evaluated in glioma patients.

#### DNA Vaccines

DNA vaccines have also been used to treat GBM. Similar to RNA vaccines, DNA vaccines in themselves act as adjuvant and antigen-encoding molecule at once. Such a DNA vaccine consists of a plasmid encoding for a specific antigen, which is usually intramuscularly administrated. This leads to transduction of DNA in myocytes and also to some extent in DCs. The presence of double stranded (ds) DNA in the cytosol of DCs induces the activation of the stimulator of interferon genes (STING) pathway via DNA sensors, e.g., DNA-dependent activator of interferon (IFN) regulatory factors (DAI) and cyclic GMP-AMP synthase (cGAS), which leads to activation of interferon regulatory factor (IRF) 3 and hence, the expression of type I IFNs. In parallel, type I IFNs are additionally induced, because unmethylated CpG DNA is bound by TLR9, which through the signaling of myD88 activates IRF7. This signaling cascade ultimately leads to the upregulation of the antigen presenting machinery, such as the expression of MHC class II and MHC class I, on which the antigens are presented to cytotoxic T cells. Transfection of myocytes also induces a similar cascade as DCs can pick up the myocyte-expressed antigens through phagocytosis as these antigens get secreted or released following apoptosis. In DCs, these antigens are processed and presented on MHC class I to cytotoxic T cells via cross presentation, a process that is further enhanced by type I IFNs. DNA vaccination has become an attractive immunotherapeutic approach against cancer, because such vaccines are very cost-effective and can be easily administered repeatedly due to their stability and safety. Results from numerous clinical trials have shown that DNA vaccines are well tolerated without induction of major adverse events. However, as with most tumor associated antigen specific vaccines, DNA vaccines often fail to induce potent immune responses as a result of immune tolerance [31].

In GBM patients, multiple DNA vaccine clinical trials have been initiated and completed. One example for a tumor-associated antigen specific DNA vaccine, which has been tested in a phase 1 clinical trial in patients with operable recurrent GBM, is the VXM01 pilot study. This study investigated safety, tolerability, and immune response to the VXM01 DNA vaccine, targeting vascular endothelial growth factor receptor 2 (VEGFR2) (NCT02718443). VXM01 is a first in kind oral DNA vaccine which has been developed for patients with advanced pancreatic cancer originally. Oral administration exploits the large surface area of the intestine to deliver the vaccine to the Peyer’s patches. The vaccine is based on life attenuated *Salmonella typhi* Ty21a bacteria carrying an expression plasmid encoding VEGFR2. VEGFR2 is expressed in tumor vasculature and has been targeted via anti-angiogenic intervention in various cancer entities, such as with bevacizumab. VXM01 was first tested in a randomized, placebo-controlled phase I clinical trial in pancreatic cancer patients, where it was found to be safe and immunogenic. Both humoral and effector, but not regulatory, T cell responses were induced by the vaccine. Vaccine-induced effector T cell responses were associated with anti-angiogenic activity, signified by reduced tumor perfusion and serum biomarkers, and with high numbers of anti-VEGFR2 effector T cells at baseline. This led to the assumption that pre-existing memory T cells play a role for anti-angiogenic activity [32, 33]. A follow-up study demonstrated that monthly boost vaccinations were able to significantly enhance and prolong specific T cell responses [34]; hence, this prime-boost schedule was administered in following trials. In GBM patients, the VXM01 pilot study demonstrated safety and immunogenicity, the VXM01 vaccine eliciting specific T cell responses in 7/12 patients and correlating with increased T cell infiltration in post-treatment tumor tissue. In terms of clinical efficacy, 58% of patients presented with OS of more than 12 months. Three patients additionally received the checkpoint inhibitor nivolumab, which led to complete response in one of these patients [35]. Based on these results, a follow-up phase 1/2 combination study evaluating safety and efficacy of VXM01 in combination with the anti-PD-L1 checkpoint inhibitor avelumab has been initiated and is currently recruiting 30 patients with relapsed GBM (NCT03750071).

As with other vaccine modalities, tumor-associated antigen-specific DNA vaccines are prone to tolerance, limiting efficient immune responses. Consequently, neoantigen-specific DNA vaccines have been developed. Only one such vaccine is under clinical evaluation in glioma patients. A phase I clinical trial assesses safety, feasibility, and immunogenicity of a personalized neoantigen-specific DNA vaccine in patients with newly diagnosed, unmethylated GBM. Feasibility will be measured by the ability to identify tumor-specific neoantigens, to manufacture a neoantigen-based DNA vaccine, and to administer the vaccine to a patient in a timeframe of four weeks post radiotherapy completion (NCT04015700). A key feature of this vaccine trial is the use of the so-called CELLECTRA^®^2000 device, which is a system to enhance the uptake and expression of plasmid-based biologicals in order to enhance vaccine efficacy, and the administration of plasmid-encoded IL-12. This trial is currently recruiting with results expected in 2023.

#### Dendritic Cell Vaccines

More experience has been gained for DC vaccines in GBM, with first clinical attempt more than 20 years ago [36]. Here, specific peptide targets or — which is mostly used — patient-autologous tumor lysates are used to load ex vivo generated and expanded autologous DC before these are re-infused to the patient. The latter is being investigated e.g., in a large phase III trial, aimed to enroll 348 patients, comparing the so-called DC-Vax-L to PBMC placebo injections. Although interim analyses have been published, treatment and follow-up are ongoing and remain blinded; first results are expected end of 2022 (NCT00045968) [37]. In the DC vaccine ICT-107, DCs are loaded with six tumor-associated antigens (TAA). This vaccine has been tested in a phase II randomized double-blind placebo-controlled trial in patients with newly diagnosed GBM (NCT01280552) [38]. This is the first, well-controlled, moderate-sized randomized clinical trial in this population showing a possible clinical benefit for a DC vaccine to date, as PFS, but not OS, was significantly increased in the HLA-A2+ treatment population, albeit at only moderate clinical relevance. In this study population, strikingly, MGMT-promotor methylated HLA-A1+ patients seemed to experience an OS benefit that was more pronounced than the PFS benefit of HLA-A2+ patients. A phase III trial in HLA-A2+ patients, however, was prematurely stopped because of insufficient financial resources to complete it [39]. DCs can be administered intravenously, intradermally, intranodally, and intratumorally. As of now, there is no consensus which route of DC administration is to be preferred [40]. In contrast to peptide, DNA, or RNA vaccines, DCs can be loaded with patient-individual lysates, therefore displaying multiple patient-individual epitopes that are often unknown. Whereas complex and time-consuming epitope selection platforms can be avoided, immune monitoring of therapy-induced T cell clonotypes remains challenging. As DC products need to be manufactured under GMP conditions and applied vitally, production costs are higher compared to RNA or peptide vaccines. However, DC activation, which is naturally suppressed in cancer patients [41, 42] but important for vaccine-induced T cell priming, can be robustly achieved in vitro.

### Peptide Vaccines

Peptide vaccines represent the traditional vaccines, as peptides are produced fast and cost-effectively. As outlined above, neoantigens are to be preferred over tumor-associated targets, while shared antigens are less laborious to produce than vaccines targeting newly discovered targets within individualized concepts. Here, we will highlight three examples of shared neoantigen peptide vaccines and exemplify successful strategies for individualized concepts including warehouse as well as newly identified vaccine targets.

#### Shared Neoantigen Targets

##### IDH1

One of the most prominent examples of shared driver mutations in neuro-oncology, with an extremely high prevalence in gliomas, are mutations in the genes encoding isocitrate dehydrogenase (IDH) types 1 and 2. *IDH* mutant tumors represent the majority of WHO grade 2 and 3 gliomas, constituting a distinct entity since the revised WHO classification in 2016 [43]. This is due to the accompanied alteration of the methylation profile via the production of 2-hydroxyglutarate (2-HG) by the neomorphic enzymatic function of mutant IDH, which leads to genomic instability and thereby qualifies *IDH* mutations as tumor-initiating true driver mutations. *IDH* mutations have been targeted by small molecule inhibitors, which are designed to inhibit the 2-HG production and currently are tested in patients with IDH-mutated glioma in four clinical trials (NCT03343197, NCT04056910, NCT02073994, NCT04195555). The vast majority of *IDH* mutant gliomas harbor the arginine to histidine substitution at residue 132 of IDH1 (IDH1R132H). We and others have demonstrated that IDH1R132H harbors an immunogenic neoepitope that is presented on MHC class II within glioma tissue, leads to spontaneous CD4+ T cell responses in some patients with recurrent IDH1R132H mutant gliomas detectable in peripheral blood, and can be targeted by peptide vaccination, which elicits a tumor-specific T helper cell response that is capable of controlling IDH1R132H-expressing tumors in an MHC-humanized preclinical model transgenic for the human MHC class II allele HLA-DR1 [19, 44, 45]. In this context, mutation-specific CD4+ T cells were shown to infiltrate the tumor and to be required for clinical efficacy, most likely by releasing proinflammatory cytokines such as interferon-γ (IFNG) and tumor necrosis factor-α (TNFA) [19, 46]. In line with strict tumor-specificity of the IDH mutation, there is no evidence of off-target toxicity of an IDH1R132H-specific vaccine from preclinical nor clinical studies. In principle, the antigenic function of IDH1R132H applies to other tumor entities harboring this mutation, such as cholangiocarcinoma, osteosarcoma, and acute myeloid leukemia, although this has not yet been formally proven. Similarly, evidence that any of the rarer IDH type 1 or 2 mutations, which occur more frequently in these tumors compared to glioma, elicit similar mutation-specific immune responses has not yet been gained. As of July 2022, three different IDH1-directed mutation-specific peptide vaccines — including one peptide-loaded DC vaccine — have been, or are currently being tested in four phase I clinical trials. Safety and preliminary immunogenicity data from the RESIST trial, investigating a 25 amino acid IDH1R132H peptide vaccine combined with a standard of care tetanus diphtheria toxoid vaccine in recurrent WHO grade 2 IDH1R132H-positive glioma, demonstrate the absence of unacceptable toxicity in all 24 evaluable patients, with 4/24 patients experiencing severe adverse events (SAE), and a frequency of 43% (9/21 evaluated patients) of peripheral T cell responses (NCT02193347). The multicenter first-in-man phase I trial of the Neurooncology Working Group (NOA) of the German Cancer Society evaluated a 20 amino acid IDH1R132H vaccine, using Montanide and imiquimod as adjuvans, integrated into adjuvant temozolomide chemotherapy in newly diagnosed grade 3 and grade 4 IDH1R132H-positive astrocytomas (NOA-16, NCT02454634). Available data from this trial demonstrate safety and immunogenicity, meeting its primary endpoints, without treatment-related SAE, and with vaccine-induced immune responses in 93.3% (30/32 evaluable patients) of patients across multiple HLA alleles [47]. In this cohort, 3-year progression-free survival (PFS) was 63%, overall survival (OS) was 84%. Of note, patients with immune responses showed a 2-year PFS of 82%. Evidence for biological efficacy of the vaccine comes from several concomitant data, demonstrating a positive correlation of intratumoral IDH1R132H peptide presentation in the baseline tumor tissue with the magnitude and sustainability of specific peripheral T cell responses, a pseudoprogression frequency of 37.5% compared to 16.7% in a molecularly matched control cohort, while pseudoprogression was exclusive to patients with immune responses and associated with increased vaccine-induced peripheral T cell responses, and the presence of infiltrating IDH1R132H-reactive T cells in a pseudoprogressive lesion. Such T cells were shown to be clonally expanded and to exhibit an activated gene expression signature. The results demonstrate that a peptide vaccine is able to induce tumor-specific T cells inside a CNS tumor. They not only provide the rationale for a phase II clinical trial, but also form the basis for rational combination strategies to enhance the infiltration and intratumoral activity of IDH1R132H-reactive T cells induced by vaccines, such as combination with ICI as in the recruiting phase I 3-arm randomized trial investigating the IDH1R132H vaccine in combination with the ICI Avelumab in recurrent IDH132H positive glioma (NCT03893903; see the “Combination with Immune Checkpoint Inhibition” section). To our knowledge, it remains unknown whether the induction of tumor-specific peripheral T cells in general can be used as biomarker for treatment response. However, in NOA-16, in particular those patients with sustained and transient peripheral immune responses had favorable clinical courses [47]. Therefore, it is tempting to speculate that robust peripheral T cell responses prior to CNS T cell invasion are required for response to therapy.

##### Histone H3

Eighty percent of diffuse midline gliomas are signified by mutations in histones H3.1 and H3.3, the majority of which present with an amino acid substitution of lysine to methionine at residue 27 (H3K27M). Similar to *IDH* mutations, H3K27M mutations are an early event in gliomagenesis, making them both clonal and tumor-specific, hence ideal targets for immunotherapy. Moreover, while the majority of H3K27M-mutated gliomas occur in pediatric patients, this mutation is a recurrent event also in midline and infratentorial gliomas of young adults and in these patient populations associated with poor outcome and resistance to alkylating chemotherapy [48–51]. The identification of H3K27M mutant variants has — like *IDH* mutations — led to a revision of the WHO classification of CNS tumors [43]. In general, H3 mutations are associated with a distinct global DNA methylation pattern [52, 53] and have neuroanatomical specificity [53–56]. In in silico and preclinical studies, we have demonstrated that mutated H3K27M peptides are presented on human MHC class I and II within tumor tissues to stimulate proinflammatory mutation-specific CD8+ cytotoxic and CD4+ T helper responses, which are detectable in the peripheral blood of patients spontaneously, and that an H3K27M-specific peptide vaccine is effective against H3K27M-mutant tumors in the above-mentioned MHC-humanized preclinical model [57]. As of July 2022, three ongoing clinical trials investigate H3K27M mutant-specific vaccines in diffuse midline gliomas (DMG), whereas one is underway. Of note, due to lessons learned from other vaccine trials for glioma, and the immunological coldness of DMG, especially regarding extremely sparse T cell infiltration [57], all but one trial are conducted in combination with ICI (see also the “Improving Vaccine Efficacy” section). A three-arm phase I clinical trial tests the safety (number of patients experiencing treatment-related AEs), efficacy (OS at 12 months), and immunogenicity of an H3.3K27M-specific short peptide vaccine, combined with the tetanus toxoid peptide and using Montanide and poly-ICLC as adjuvans, previously shown to elicit CD8-driven T cell responses in experimental models, in pediatric patients with newly diagnosed DMG or other glioma in combination with the ICI nivolumab in one arm (NCT 02,960,230) (see also the “Improving Vaccine Efficacy” section). Notably, inclusion criteria are restricted to HLA-A2 and H3.3K27M positive patients. Available clinical data show that administration of the H3.3K27M vaccine was well tolerated with no grade IV treatment-related adverse events [58]. Another phase I trial designed as a 3 + 3 dose escalation plus expansion trial evaluates safety and tolerability as dose-limiting-toxicity outcome measure of incremental combination of a so-called rHSC-DIPGVax vaccine with ICI balstilimab (anti-PD-1) and zalifrelimab (anti-CTLA-4). rHSC-DIPGVax contains an immunostimulatory heat shock protein combined with 16 neoantigenic peptides found in the majority of diffuse intrinisc pontine gliomas (DIPG) and DMG, presumably including H3K27M, in newly diagnosed DIPG and DMG (NCT04943848). The ENACTING trial is designed as a 6 + 3 dose escalation trial to assess the maximum tolerated dose as well as safety (number of AEs and 1-year survival rate), efficacy (PFS and OS), and immunogenicity as determined by IFN-γ ELIspot assays of peripheral blood mononuclear cells, of an H3.3K27-specific short peptide vaccine together with the adjuvant poly-ICLC, in 30 HLA-A2 positive children with DIPG (NCT04749641). The not-yet-recruiting INTERCEPT-H3 phase I trial (at the time of publication of this article) plans to administer a long peptide vaccine containing a K27M-mutated histone-3 sequence with Montanide and imiquimod as adjuvant in combination with the ICI atezolizumab to 15 adult patients with newly diagnosed H3.1K27M or H3.3K27M mutant DMG (NCT04808245). Primary outcome measures are safety and tolerability (endpoint regime limiting toxicity (RLT)), as well as immunogenicity (presence of H3K27M-specific T cell responses in peripheral blood), secondary parameters include efficacy as determined by PFS and overall response rate (ORR), as well as the association thereof with immunogenicity. Importantly and uniquely, vaccination is started with standard radiotherapy (RT), while Atezolizumab application starts after completion of RT.

##### EGFRvIII

A prominent shared neoepitopic target expressed in 20–30% of GBM is the epidermal growth factor receptor variant III (EGFRvIII) mutant. It is generated by alternative splicing of exons 2–7 — representing the ligand-binding domain — with subsequent generation of a neoepitope by fusion of exon 1 with exon 8. Functionally, EGFRvIII is constitutively active in the absence of epidermal growth factor (EGF), leading to enhanced proliferation and inhibition of apoptosis. Driving malignancy, this tumor-specific antigen has been very attractive in GBM. More than 20 clinical trials targeting EGFRvIII, incl. vaccines, small molecule inhibitors, and antibodies, have been registered on clinicaltrials.gov, e.g. NCT02573324, NCT01520870, NCT02101905. As for IDH mutations, single tumor cells usually carry one mutated and one wildtype (wt) copy. However, in contrast, tumors display intratumoral heterogeneity, EGFRvIII being only expressed in tumoral subclones [59]. Consequently, although the EGFRvIII-specific peptide vaccine Rindopepimut^®^ induced robust anti-EGFRvIII antibody responses and CD8+ T cell responses in patients with EGFRvIII-positive tumors, the vast majority of tumors of patients in the first phase I/II clinical trial in 2015 who experienced recurrence had lost EGFRvIII expression [60]. Interpreted as immune escape after biological vaccine efficacy, the randomized double-blinded phase III ACT-IV trial was initiated to test the efficacy of rindopepimut^®^ compared to control in addition to chemotherapy with temozolomide in patients with newly diagnosed EGFRvIII-positive glioblastoma after maximal surgical resection (NCT01480479). 745 patients were enrolled, of which 405 had minimal residual disease (MRD), 371 received rindopepimut^®^ (195 with MRD) and 374 received placebo (210 with MRD). This trial was discontinued after the interim analysis by the independent Data Safety and Monitoring Board showed no significant benefit of the vaccine on overall survival for patients with MRD, the primary outcome of the trial [61, 62]. The failure of this phase III study has gained broad attention in the community, highlighting the importance to carefully interpret results of preceding phase II trials, understand the mechanisms, and draw firm conclusions for future rationale design of — potentially combinatorial — immunotherapeutic approaches [63–65]. Unfortunately, ACT-IV did not provide evidence for vaccine-driven antigen loss at recurrence as a surrogate for biological activity, as it has been previously demonstrated that EGFRvIII expression is spontaneously lost in 50% of recurrent GBM [66], a rate that was observed in the ACT-IV independent of treatment arm. Mechanistically, preclinical studies had suggested that rindopepimut^®^ may be effective due to antibody-dependent cellular cytotoxicity (ADCC), suggesting that an antigen-dependent T cell response is dispensable [67]. However, the therapeutic efficacy of cancer vaccines is usually mainly mediated by induction of cellular immunity [68]. Yet all ACT studies have failed to analyze potential EGFRvIII-specific vaccine-induced T cell responses and to assess signs for intratumoral immunoreactivity in recurrent tissue. In an exploratory analysis, 2-year survival rate was increased by rindopepimut^®^ compared to control in patients with significant residual disease. It is tempting to speculate that this may hint towards a beneficial neoadjuvant immunotherapeutic intervention in contrast to the adjuvant setting, as has been shown for peripheral solid tumors such as melanoma [69]. Whether the failure of ACT-IV can be attributed to concomitant temozolomide remains to be determined. Interestingly, in the double-blinded phase II ReACT trial, assessing safety, 6-months PFS, PFS, OS, and ORR of rindopepimut^®^ plus the vascular endothelial growth factor (VEGF)-neutralizing antibody bevacizumab versus bevacizumab plus control in 73 cases of relapsed EGFRvIII-positive GBM, demonstrated a prolonged OS by rindopepimut^®^ (NCT01498328) [70]. Here, high antibody titers seemed to correlate with prolonged OS. If — and how — the addition of bevacizumab led to an enhanced efficacy in the ReACT trial remains speculative. VEGF inhibition may lead to the reversion of tumor-associated immunosuppression and thereby enhanced T cell priming and / or ADCC [63]. In addition to targeting EGFRvIII by a peptide vaccine, EGFRvIII-specific CAR T cells have been designed and evaluated in several clinical trials (see the “CAR T Cell Therapy” section).

#### Individualized Peptide Vaccines

Given that gliomas are characterized by only few shared antigens but rather a variety of an individual mutational and genetic overexpression landscape, the development of individualized peptide vaccine concepts is an important pillar in targeted glioma immunotherapy in order to maximize potential patient populations and clinical benefit. Conceptually, such individualized vaccines may be administered also in combination with those targeting shared vaccines; however, they constitute an important option for patients whose tumors do not express shared antigens. Notably, the most malignant and most frequent glioma, GBM, hardly expresses any shared mutated antigens, one exception being EGRvIII, hence will probably benefit the most from individualized concepts. There are two levels of individualization: (i) a set of previously identified antigens and corresponding peptides is defined and manufactured upfront as a warehouse, while patient tumors and, if required, peripheral blood, are analyzed during the molecular screening phase of recruitment for gene expression, and possibly antigen presentation and individual immunogenicity, respectively, of such targets. Based on these data, targets are selected for the individual patient. Due to the nature of pre-defined antigens, these constitute mostly TAA. (ii) In a truly individualized setting, novel antigens are identified in every tumor, mainly based on mutanome data generated by mutation discovery analysis, as well as gene expression data. These antigens are therefore mostly neoantigens. Corresponding antigen presentation and immunogenicity analysis have to be integrated prior to manufacturing and administration. As all analyses must be completed prior to administration, they prolong the time window until treatment start by months.

##### GAPVAC Trial

In order to effectively utilize this prolonged time window for GBM patients, for whom weeks are critical, the phase I clinical GAPVAC trial combined both individualization levels in two treatment phases, and integrated the resulting highly individualized vaccinations into SOC treatment [11]. In the first phase, actively personalized vaccine (APVAC) 1, appropriate antigens were selected from a premanufactured warehouse of 39 pre-defined unmutated HLA class I-binding antigens, 11 of which had previously been administered as a multipeptide vaccine (IMA950) in a phase I clinical trial for newly diagnosed GBM [71]*.* In the previous trial, the IMA950 vaccine was well-tolerated and elicited immune responses in 90% (36/40) of patients, of which 56% (20/36) were multi-peptide responders. Notably, in this trial, patients were randomized into two cohorts; one receiving the first three vaccinations prior to SOC radiochemotherapy onset, while in the other cohort, vaccinations started at least one week after completed SOC cycles. While there were no differences in number of responders between both cohorts, responses seemed to be more sustained in patients receiving vaccination after SOC. Yet, such differences were not reflected in differential OS [71]. In the GAPVAC trial, tissue was screened for presentation on HLA and RNA expression of the 39 warehouse antigens, while peripheral T cells were tested for pre-existing antigen-specificity via multimer staining. Vaccination with 7 HLA class I antigens and 2 HLA class II antigens selected for each patient, and a vial marker peptide, using poly-ICLC and granulocyte–macrophage stimulating factor (GM-CSF) as adjuvant started six weeks after completion of SOC radiochemotherapy and during the first maintenance temozolomide (TMZ) cycle. For APVAC 2, the tumor mutanome was defined from the same tumor tissue using peripheral blood cells as germline reference. As for APVAC 1, presentation of these newly identified mutated epitopes was assessed; however, none of the identified mutations were detectable in such peptidomes, highlighting the challenge to detect neoepitopes on HLA molecules even by high-sensitivity mass spectrometry. Overall, the era of locoregional cell delivery and molecular safety switch technologies, as well as the procedural and technical limitations to identify neoepitopes by HLA ligandome analyses, might lead to a renaissance of glioma-associated antigen targeting in the near future. In GAPVAC, as a second and third strategy, epitopes were selected based on HLA binding and immunogenicity in silico predictions, or unmutated HLA class I epitopes not part of the APVAC 1 warehouse were selected. 11/15 patients who received APVAC 1 vaccinations received APVAC 2 vaccinations with 1 or 2 peptides, of which one was unmutated in 5 cases, approximately 12 weeks after start of APVAC 1 vaccinations, leading to a temporal overlap of APVAC 1 and 2 administrations. Primary endpoints of the GAPVAC trial were safety, tolerability, and immunogenicity of vaccinations, as well as feasibility of the concept, all of which were met. As for feasibility, 16% (9/58) patients screened could not be enrolled due to limitations of GMP peptide production capacity, which summed up to 1–2 patients per month. Further optimization of the process by reducing complexity and duration may be required to reduce dropout due to limitations in the manufacturing process. Of 15 patients receiving vaccinations, all experienced drug-related adverse events, as expected, while 2 patients experienced an anaphylactic reaction after vaccinations, and one required high-dose steroids to relieve a potentially immune-related grade 3 brain edema. In 92% of patients, APVAC 1 induced CD8+ T cell memory responses which lasted several months. As exemplified by PTPRZ1- and NLGN4X-specific T cells, their T cell receptors (TCR) were able to license transgenic T cells to exhibit cytotoxic activity towards target-expressing tumor cell lines, confirming natural processing within the tumor cells. 69% of patients receiving HLA class II-restricted vaccinations demonstrated CD4+ T cell responses, which were mainly T helper responses. Neoepitope APVAC 2 vaccinations induced CD4+ multifunctional T helper cell responses in 80% of patients, some of which were accompanied by CD8+ T cell responses. In contrast, only 1/6 unmutated APVAC 2 epitopes elicited a CD8+ immune response, highlighting a necessity for prior immunogenicity testing of HLA class I epitopes, which was not performed in APVAC 2. Among 15 patients who received vaccination, median OS was 29.0 months and median PFS was 14.2 months; OS of 11 patients exceeded the median OS for SOC-treated GBM of 14 months. Of note, one patient who exhibited favorable T cell responses to APVAC 1 and 2, including combined CD4 and CD8 T cell responses to an APVAC 2 epitope and T helper responses to both HLA class II APVAC 1 epitopes, experienced an OS of more than 39 months. Strikingly, APVAC 1 HLA class II epitope-reactive CD4+ T cells could be detected in re-resection tissue as long as 26.8 months after diagnosis and almost 10 months after the last APVAC 2 and 13 months after the last APVAC 1 vaccination. In the context of epitope selection during personalized vaccine trials, the GAPVAC trial on the one hand provides arguments for exploitation of HLA class I epitopes due to the low TMB in glioma as wells as the failure of neoantigen detection on HLA class II tumor ligandomes and the lack of reliable HLA class II binding prediction algorithms. However, from another perspective, these data demonstrate the huge versatility and exploitability of HLA class II antigens in several ways: (i) as HLA class I epitopes are very restricted to a certain HLA-type, such specific HLA type is an important inclusion criterion. In the GAPVAC trial, 20/58 patients had to be excluded due to non-suitable HLA-types. In contrast, class II epitopes often bind several HLA types, as has been demonstrated e.g., for the IDH1R132H epitope [47]. (ii) Additionally, potential HLA class I epitopes, in contrast to class II epitopes, often present with a lack of immunogenicity, hence require prior immunogenicity testing and demonstrate a lower hit rate. Strikingly, only 50% of pre-selected unmutated class I epitopes elicited responses after vaccination, while 85% of mutated class II epitopes, selected solely by expression, did so, of which 45% elicited a CD8 response in addition. (iii) Along this line, it may be even needless to test HLA class II binding of such epitopes as in HLA class II ligandomes. The failure to detect mutations on HLA class II ligandomes from tumor tissues may be due to limited sensitivity, while presentation of these epitopes may predominantly take place in the periphery e.g. in draining lymph nodes, by professional APC such as dendritic cells (DC), which do not infiltrate brain tumors in high numbers. Together, these factors argue that selection of potential class II epitopes is not required to be further curtailed. (iv) Class II epitopes may harbor nested CD8 epitopes in addition, while T helper responses in themselves can be multifunctional, as shown for more than 50% of responses to APVAC 2 in the GAPVAC trial, which may enhance clinical benefit.

##### NeoVax Trials

Another individualized peptide vaccine approach exclusively applies neoepitopes in a multi-peptide vaccine. The so-called personalized NeoAntigen Cancer Vaccine (NeoVax) was originally designed for late-stage melanoma patients, where it targeted 13–20 neoantigens per patient, which were detected and selected based on comparative WES of tumor and normal cells to detect mutations, RNA-seq to verify expression levels, and HLA-A and –B binding predictions to verify antigen presentation, and administered with the adjuvant poly-ICLC (NCT01970358) [23, 72]. This phase I trial completed in 2018 met its primary endpoints assessing safety, feasibility, and immunogenicity in 10 enrolled patients, of which eight demonstrated with typical high TMB and six received five priming and two booster vaccinations with four peptide pools according to trial protocol. Adverse events were restricted to grade 1, while all patients demonstrated a favorable outcome at 25 months median follow-up. All patients developed post-vaccine poly-functional, mostly CD4+ , T cell responses which were mostly strongest after the first boost, mostly mutation-specific, and inducible by endogenous presentation of antigen by either minigene-expressing DC or autologous melanoma cell lines in 2 cases. Of note, both patients with more malignant stage IV disease demonstrated the lowest immune responses and experienced recurrence, after which they received the checkpoint inhibitor pembrolizumab, leading to complete response. Months later, both patients showed sustained, but also repertoire-broadened T cell responses. Interestingly, gene expression profiles of post-vaccine antigen-specific CD4+ T cells from two vaccine-responding patients demonstrated a transition from naïve to effector and memory functions [23]. In a follow-up analysis after a median of four years after NeoVax treatment, incl. two additional patients, all patients were alive, with 6/8 patients disease-free [72]. Persistent neoantigen-reactive T cells were detected in the periphery and demonstrated memory phenotypes, while emergence of multiple neoantigen-specific TCR clones suggested diversification of the repertoire. Additionally, neoantigen-specific T cells as well as epitope spreading were observed in recurrent tumor tissue.

These encouraging results led to the application of NeoVax in multiple entities, including glioma. The first trial investigating NeoVax specifically in glioblastoma was designed as a pilot study in which NeoVax was to be combined with nivolumab or nivolumab plus ipilimumab in different schedules in five different cohorts of patients with newly diagnosed unmethylated GBM (NCT03422094). It aimed to investigate how the timing of immune checkpoint blockade combined with vaccine affects clinical and immunological response. Because CTLA-4 has a role in early priming and PD-1 in later local tissue response, sequential administration of different checkpoint blockers which target these separate pathways was hypothesized to synergistically boost the anti-tumor immune response. However, this trial was terminated after recruitment of only three patients due to a change of focus towards cell therapy. Whether limited recruitment played a role for termination remains speculative. The only recruiting trial applying NeoVax in GBM patients investigates its feasibility, safety, and tolerability in patients with newly diagnosed GBM in a phase 1 (NCT02287428) [12]. Originally, NeoVax was following standard radiotherapy. After completion of accrual, four additional cohorts were added, in which pembrolizumab is additionally applied at varying schedules. The checkpoint blockade may be administered with radiotherapy and / or starting with NeoVax, which is administered after radiotherapy in each cohort. One cohort additionally receives standard chemotherapy with concurrent and adjuvant temozolomide (TMZ). The purpose of adding these additional cohorts is to determine optimal treatment regimen for combination of a vaccine with checkpoint inhibition. Results are yet awaited long term and expected no earlier than 2026.

### Improving Vaccine Efficacy

#### Combination with Immune Checkpoint Inhibition

With the advent and success of immune checkpoint inhibition (ICI) in various tumor entities, especially peripheral solid tumors, this immunotherapy approach has gained the most popularity within cancer immunotherapy. This has led to massive attempts to translate clinical success to glioma patients. However, ICI has failed to improve survival in unselected glioma patient cohorts yet providing evidence that neoadjuvant ICI is associated with intratumoral inflammation and favorable outcome. Moreover, specific biomarkers have been shown to potentially be predictive also for specific glioma subtypes. These developments have led to multiple clinical trials investigating ICI combination studies, adding such inhibitors to multiple types of vaccines. As outlined above, running vaccine trials often add ICI antibodies to the treatment regimen. First results of these combinatorial treatments hinted towards clinical benefit of such combinations and demonstrated a toxicity profile similar to ICI monotherapy. The scientific rationale to combine ICI with cancer vaccines is the hypothesis that ICI can amplify vaccine-induced T cell responses within the tumor microenvironment, inhibiting immune evasion and providing reinvigoration of exhausted T cells. Numerous preclinical studies in cancer models show that immune checkpoint inhibitors synergize with cancer vaccines by amplifying vaccine-induced T cell responses [73, 74]. Antonios et al. observed an increase of T cells expressing memory and tumor homing markers in experimental GL261 gliomas following anti-PD-1 therapy in combination with DC vaccination [74]. Such synergies have also been demonstrated in clinical studies [75–78]. For instance, a phase 1 study of nivolumab plus peptide vaccine in resected stage IIIC/IV melanoma patients demonstrated “statistically significant increases in melanoma antigen-specific CD8+ T-cell populations and decreases in PD-1 expressing T-cells with exposure to nivolumab and vaccine” [75]. Although the ICI antibody avelumab, targeting programmed death ligand 1 (PD-L1), has failed to improve OS or PFS in clinical trials treating newly diagnosed and recurrent glioblastoma patients when administered as monotherapy in addition to standard of care, two clinical trials are currently investigating avelumab vaccine combinations. One of those administers VXM01 to patients with progressive glioblastoma (NCT03750071), while the AMPLIFY-NEOVAC trial combines the IDH vaccine with this type of ICI in a neoadjuvant setting in of window-of-opportunity phase 1 clinical trial (NCT03893903). This trial evaluates safety and immunogenicity of the IDH vaccine alone or in combination with avelumab in patients with recurrent and re-resectable IDH1R132H mutant glioma, comparing these two arms to an avelumab monotherapy arm. This trial is the first ICI clinical trial in patients with IDH1R132H mutant glioma that applies ICI in a neoadjuvant setting. This design may be more effective as suggested by trials using neoadjuvant ICI in recurrent glioblastoma [79] and at the same time allows detailed molecular and immunological analysis of post treatment tumor tissue. Consequently, immunogenicity assays are not only aimed at peripheral blood responses, but at intratumoral specific T cell characterization, including functionality and clonality. Exploratory analyses will determine efficacy and aim to identify predictive molecular immune and imaging biomarkers, such as presentation of the IDH1R132H epitope within the pre-treatment tumor tissue, and tumor microenvironmental molecular and transcriptomic profiles [80]. Similarly, H3K27M vaccines will be applied in combination with the ICI Atezolizumab or Nivolumab to patients with newly diagnosed H3 mutant DMG in the INTERCEPT-H3 trial (see above) and another phase 1/2 trial (NCT02960230), respectively.

Conceptually, it has been a matter of debate which immune checkpoint to target in glioma, whether or not the inhibitor is required to cross the blood–brain-barrier, depending on whether the checkpoint is expressed on tumor or T cells. In glioma clinical trials, the most used ICI target PD1 or PDL1 on T cells or tumor cells and antigen presenting cells, respectively. CTLA4 is an early immune checkpoint acting during T cell priming by binding to CD80 and CD86 on antigen-presenting cells, inhibiting T cell activation and proliferation. Hence, CTLA4-specific antibodies mainly act within the lymph nodes, while PD1- and PDL1-specific ICI act within the tumor microenvironment during a later stage of T cell activation. Of note, although multiple drugs have shown to fail to cross the blood brain barrier, ICI antibodies have been shown to be able to penetrate the brain as cargo on the respective target cells. Reasons for ICI failure for glioma patients, such as the unique immune microenvironment in the brain, impact of corticosteroids, and inter-and intra-tumoral heterogeneity, as well as an overview on past and current clinical trials applying ICI alone or in combination with radiotherapy or bevacizumab to distinct glioblastoma subgroups, which may shed light on predictive markers for ICI efficacy, have been extensively reviewed elsewhere [5].

#### Combination with Small Molecule Mutant IDH Inhibition

A more specific combination is the application of small molecule mutant IDH inhibition with the IDH vaccine. As outlined above, the IDH mutations lead to the production of 2-HG by a neomorphic enzymatic function, leading to genomic instability. IDH mutant glioma have been shown to be dependent on the maintenance of this mutation, one reason why the IDH mutations are maintained upon progression and recurrence. On this basis, IDH mutant inhibitors have been developed and are currently tested in clinical trials. Pre-clinically, we and others have shown that mutant IDH not only affects tumor cells, but also infiltrating cells of the tumor microenvironment. One such mechanism is the altered molecular signature of tumor cells, which leads to distinct chemokine landscapes and thereby influences immune cell infiltration and function. On the other hand, 2HG directly affects glioma infiltrating T cells and myeloid cells, inhibiting T cell function and proliferation via altered ATP synthesis, NFkB signaling, and ornithine metabolism. In myeloid cells, 2-HG induces complex re-orchestration of tryptophan metabolism, which results in activation of the aryl hydrocarbon receptor [AHR]. AHR is known to have an immunosuppressive function in antigen-presenting cells via the induction of a variety of target genes. Consequently, we and others have shown in pre-clinical IDH mutant glioma models that mutant IDH or AHR inhibition via small molecule inhibitors has synergistic antitumoral effects when combined with immunotherapy such as ICI or vaccination [81–83].

Since the discovery of IDH mutations and their impact on tumor cell biology, several mutant IDH inhibitors have been developed, some of which have been under clinical investigation. These inhibitors are able to significantly reduce 2-HG levels and via changes in DNA methylation lead to re-differentiation of IDH mutant tumor cells in vitro and in mouse tumor models [84]. Anti-tumoral activity has been demonstrated in such models. As IDH mutations also frequently occur in acute myeloid leukemia (AML), although mostly affecting IDH2, some of these inhibitors have also been tested in AML samples and models. For IDH mutant glioma patients, several phase 1, mostly dose escalation clinical trials have been initiated and in part been completed. In those trials for which results have been reported so far, IDH inhibitors such as vorasidenib (AG-881), ivosidenib (AG-120), and BAY1436032 demonstrated safety, brain penetrance, and target inhibition. Importantly, evidence for clinical activity and objective response in some IDH mutant low-grade glioma patients has been reported (NCT02481154, NCT02746081, NCT03030066, NCT03343197) [85–89]. Interestingly, one study preliminarily reported beneficial effects on the tumor immune microenvironment, including an increase in CD3+ and CD8+ TILs, and upregulation of type I interferon signaling and antigen presentation [89]. Based on the encouraging results using vorasidenib in recurrent or progressive glioma, demonstrating responses in some patients, a phase 3 randomized clinical trial testing vorasidenib versus placebo has been initiated (NCT04164901). This so-called INDIGO study evaluates anti-tumor activity of vorasidenib in an early stage of disease in patients with recurrent grade II, non-enhancing IDH mutant glioma, who are treated with surgery only. This approach aims to substitute the wait-and-watch approach following surgery in patients with low-risk low-grade glioma, avoiding alkylating chemotherapy- and radiation-associated toxicity.

Ivosidenib is the first and only IDH small molecule inhibitor that is currently under clinical investigation in combination with immunotherapy in IDH1 mutant tumors, including gliomas. A phase II single arm trial evaluates safety, response rate, progression-free and overall survival of 35 patients with advanced solid tumors, i.e., non-resectable or metastatic, or enhancing gliomas treated with ivosidenib in combination with the ICI Nivolumab (NCT04056910). Similarly, one can envision, based on pre-clinical observations outlined above, that a combination of IDH inhibition and specific vaccination will show clinically meaningful synergistic effects on response. However, no such clinical trial has been registered on clinicaltrials.gov yet.

## Adoptive Cell Therapies

Vaccines require immunocompetence of cancer patients, who usually undergo massive immunosuppression during their standard chemotherapeutic treatment. In addition, especially glioma patients mostly receive steroids such as dexamethasone to relieve them of cerebral edema. Such treatment has to be stopped prior to vaccination due to its immunosuppressive nature. These and other factors, such as the immunosuppressive microenvironment maintained by glioma cells, are reasons for an often limited immunogenicity of vaccines. Cellular therapies can circumvent the requirement for immunocompetence. Instead of inducing immune responses in the patient, patient-autologous T cells are isolated, expanded and activated in culture, and re-infused. Originally, such T cells had been isolated directly from the tumor as tumor-infiltrating T lymphocytes (TIL), as it was hypothesized that these mostly include tumor-specific T cells. Although it has more recently been shown that within TIL, the most tumor-specific T cells are exhausted — which is exploited during ICI — such T cells were selected based on exhaustion as a surrogate marker for specificity and re-invigorated in culture. However, this approach harbors several drawbacks: (i) the frequency, and hence the absolute number, of truly tumor-specific T cells within the infused cellular product is unknown and can vary between patients and doses; hence, efficacy and off-target side effects might be detrimental; (ii) activation in culture alters the TCR repertoire of these cells due to variant expansion of certain clones, further exacerbating issue (i) [90]; (iii) potential incomplete in vitro reinvigoration of exhausted TIL; (iv) limited in vivo expansion capacity after preceding strong in vitro stimulation; (v) the antigenic target of such T cells is unknown, making a prediction and risk assessment of potential on-target side effects, which occur when targeted antigens are expressed in healthy tissue, difficult. Therefore, focus has shifted towards the exploitation of peripheral autologous T cells that are genetically equipped with a pre-identified and well-characterized antigen-specific surface receptor, which has mostly been designed as a so-called chimeric antigen receptor (CAR), but which can also be a natural TCR, both of which will be described by design, characteristics in antigen detection, and clinical application in glioma patients, in the following chapters.

### CAR T Cell Therapy

Chimeric antigen receptor (CAR) T cell therapy targeting the surface antigen CD19 has demonstrated to elicit clinical responses in non-solid tumors such as multiple myeloma and leukemia and has recently been approved by the U.S. Food and Drug Administration (FDA) and European Medicines Agency (EMA). For solid tumors, multiple CAR T cell clinical trials have been initiated [91]. CARs are composed of an antibody-derived extracellular recognition domain, a hinging transmembrane domain, and an intracellular TCR-derived signaling domain. The antibody-derived variable regions are able to recognize extracellular antigens, bypass antigen presentation on MHC by tumor cells or professional APC, and are independent of co-stimulation. Alternatively, modified natural ligands of surface receptors may be used as extracellular recognition domains. Second, third, and fourth-generation CARs have been developed by modifications of the intracellular signaling domain and the addition of co-stimulatory signals [92]. In preclinical studies, several CARs against glioma-associated and specific antigens have been developed, which have been under clinical investigation.

#### Glioma-Associated Antigens for CAR T Cell Therapy

##### IL13Rα2

Interleukin-13 receptor subunit alpha-2 (IL13Ra2) is a tumor-associated antigen that was the first target in GBM to be exploited for CAR T cell therapy. IL13Ra2 is highly overexpressed on tumor cells in a high frequency of GBM patients [93–95]. It binds its ligand IL13 with higher affinity than the ubiquitously expressed IL13Rα1, which enables efficient IL13Rα2 targeting with modified IL13 variants [96–98]. This fact was exploited for the development of the first IL13Rα2-specific CAR, which included a so-called zetakine composed of an extracellular altered IL13 domain and demonstrated effective tumor cell lysis in human xenograft models [99]. The first-in-human clinical trial evaluated an IL13Ra2-specific IL13-zetakine CAR T cell product in three patients with recurrent GBM injected directly into the resection cavity in 12 doses over 5 weeks [100]. Minor adverse events, including temporary brain inflammation events, were reported, indicating promising tolerability of T cell products. Indication for biological activity, yet therapy-driven antigen loss, came from a decrease in tumoral IL13Rα2 expression after CAR therapy in one patient. Current phase I clinical trials evaluate hinge-optimized, 41BB-costimulatory IL13Rα2 CAR T cell therapy for ependymoma, leptomeningeal GBM, and medulloblastoma (actively recruiting, NCT04661384), as well as recurrent or refractory malignant glioma (non-recruiting, NCT02208362). Results are expected for 2025.

##### Her2

The human epidermal growth factor receptor 2 (Her2) constitutes a GBM-associated antigen in approximately 80% of GBM patients [101, 102]. Her2-specific CAR T cells demonstrated preclinical efficacy in several tumor models, including a patient-derived xenograft (PDX) model using patient-autologous T cells to minimize allogenic reactions [102–106]. Clinical translation of Her2-specific CAR cell therapy has been hampered by a case report on one patient with metastatic colon cancer, who experienced a severe and lethal cytokine storm after administration of a Her2-directed CAR cell product, in 2010 [107]. However, subsequent clinical studies reported no severe systemic toxicities [108–111]. A phase I dose escalation study using HER2-specific CAR T cells derived from virus-specific T cells (VST) in 17 GBM patients demonstrated only limited clinical efficacy with a median OS of 11.1 months, and lack of durable expansion of HER2-specific CAR VST in the peripheral blood [110]. An interim analysis of a phase I clinical trial using HER2-specific CAR T cells recently reported highly elevated interferon-induced C-X-C motif chemokine ligand 10 (CXCL10) and CC-chemokine ligand 2 (CCL2) levels in the cerebrospinal fluid (CSF) after CAR T cell infusion as well as indications for pseudoprogression as evidenced by MRI-based detection of vasogenic edema and local intensified contrast enhancement (NCT03500991) [111]. Recently, off-the shelf NK cell line NK-92 genetically engineered to express a HER2-targeting CAR, generating so-called NK-92/5.28.z cells have been reported to specifically lyse GBM-derived cell lines and to exhibit strong specific anti-tumor effects with prolonged survival, secondary resistance to re-challenge with tumor cells, and without relevant toxicity in xenografts and multiple immunocompetent preclinical mouse models incl. the glioblastoma model GL261 [112, 113]. On this basis, the CAR2BRAIN phase 1 multicenter German-wide dose-escalation clinical trial evaluates the safety and tolerability of NK-92/5.28.z cells in patients with recurrent HER2-positive glioblastoma (NCT03383978) [114]. Further primary objectives are to determine the maximum tolerated dose (MTD) as well as persistence of infused cellular product and cytokine profiles in blood and CSF.

##### GD2

The tumor-associated antigen disialoganglioside GD2 is frequently overexpressed in neuroblastoma. CAR T cell therapy was able to abrogate tumor progression in a xenograft model and has demonstrated remarkable preclinical efficacy in PDX models of H3.3.K27M-mutated midline gliomas [115, 116]. Second-generation 4-1BB CAR T cells were able to clear tumors from different localizations with a small amount of GD2-negative tumor cells remaining, again suggesting therapy-induced antigen loss. However, severe neuroinflammation in immunodeficient mice was reported. In the first-in-human phase I clinical dose-escalation trial applying GD2-directed CAR T cells manufactured with retroviral vectors to patients with H3K27M-mutant midline gliomas such as diffuse intrinsic pontine gliomas, patients received one injection at escalating dose levels administered after cyclophosphamide/fludarabine-based lymphodepletion, followed by subsequent infusions in eligible patients who exhibited clinical benefit (NCT04196413) [117]. An interim analysis covering the first 4 patients demonstrated that despite symptoms of on-tumor neurotoxicity such as cytokine release syndrome and immune effector cell associated neurotoxicity, no on-target off-tumor side effects were detected, in spite of target expression in normal neural tissues, supporting the notion that high antigen density is required for effector function of CAR T cells [118]. Three of four patients exhibited marked improvement or resolution of neurological deficits as well as radiographic responses, while an increase in inflammatory cytokines both in CSF and plasma was observed. Mechanistic insights into cellular composition of the CSF, i.e., local immune cells, were drawn from longitudinal single cell transcriptome analysis, which revealed a marked decline of regulatory T cells (Treg) as well as a significant increase in pro-inflammatory macrophages defined by an interferon response signature, in the CSF at the peak inflammation time point [117].

##### EphA2

Ephrin type-A receptor 2 (EphA2) is considered a glioma-associated antigen with expression in healthy tissue limited to some epithelial cells [119]. It is a receptor tyrosine kinase that binds ephrin-A family ligands and has functional relevance in tumor cells as downstream signaling plays a role in migration, proliferation, differentiation, and integrin-mediated adhesion [120, 121]. Its overexpression has been linked to decreased overall survival in patients with GBM [122]. Several preclinical studies showed potent anti-tumor activity of EphA2-directed CAR T cells against glioma-initiating cells in GBM xenograft and medulloblastoma mouse models [123–125]. However, to date, clinical studies evaluating EphA2-directed CAR T cells have not yet been initiated.

##### B7-H3

B7 homolog 3 (B7-H3) is a type I transmembrane protein that is overexpressed in 76% of GBM [126]. Pre-clinical local application of B7-H3-specific CAR T cells induced durable responses in immunodeficient mice harboring the human GBM cell line U87 [127]. More recently, B7-H3 has been successfully co-targeted by B7-H3-CD70 tandem CARs (Tan-CAR) in non-glioma PDX models, improving preclinical response compared to single targeting of either antigen [128]. Of note, B7-H3 CD70 co-expression has also been reported in glioma. Currently, three clinical trials investigating safety of B7-H3-specific CAR T cells in recurrent or refractory GBM are registered, two of which are actively recruiting patients (NCT05241392, NCT04077866). Two of these trials are phase I dose-escalation trials to determine maximum tolerated dose in an open label, 1 arm design (NCT05241392, NCT05366179), while one phase 1/2 trial compares B7-H3 CAR T cell infusions in conjunction with chemotherapy using temozolomide (TMZ) to TMZ alone to test CAR T cell efficacy measured by OS (NCT04077866).

#### Glioma-Specific Neoantigens for CAR T Cell Therapy

##### EGFRvIII

As outlined above, EGFRvIII may serve as a potent target for immunotherapies. The feasibility of EGFRvIII-directed CARs has been extensively studied. Among seven EGFRvIII-directed antibodies, three have been identified to be suitable for a CAR T cell product based on the production of effector cytokines in response to EGFRvIII-expressing glioma cells [50]. Preclinical efficacy of murine third generation EGFRvIII-directed CAR T cells was shown in an immunocompetent syngeneic mouse model [129]. In contrast to studies in immunodeficient mice, two key findings could be drawn from this study. (1) Lymphodepletion is required for pre-clinical efficacy of systemically injected CAR T cells. (2) CAR T cell therapy induced long-term endogenous immunity, protecting mice from rechallenge with EGFRvIII-negative tumors. In terms of enhancing efficacy, overexpression of the micro RNA miR-17–92, a miRNA that has been reported to enhance T cell survival and interferon production and to be downregulated in GBM-infiltrating T cells, led to increased T cell function [130]. Based on a comprehensive preclinical study, 10 patients with GBM were treated with a single dose of EGFRVIII-directed CAR T cells in the first-in-human clinical EGFRvIII-CAR T cell trial [131, 132]. CAR T cells were detected within the tissue upon recurrence, which showed reduced EGFRvIII expression. However, such reduced EGFRvIII expression reflected the natural course of disease, but not antigen loss due to immunological escape [62]. Additionally, infiltration of CAR T cells was associated with an increase in T regulatory cells and inhibitory molecules such as PDL1, tumor growth factor β (TGFB), and IL10. Recent EGFRvIII-specific CAR T cell trials for GBM have been closed prior to completion due to various reasons, including toxicity, missing objective clinical responses, and shift towards combinatorial approaches (NCT01454596, NCT02664363, NCT02209376, NCT03283631). These observations, together with the limited efficacy of an EGFRvIII-specific vaccination using Rindopepimut, question the suitability of EGFRvIII as an antigen target for GBM immunotherapy.

### TCR T cell Therapy

In contrast to CAR-transgenic T cell therapy, TCR-transgenic therapy enables targeting of intracellular proteins that represent a major source of neoantigens. However, TCR-transgenic therapy depends on a functional antigen processing machinery and surface MHC class I expression. However, in gliomas, tumoral MHC class I loss is rare. Despite their local adaption to immunosuppressing phenotypes, blood-borne macrophages represent a continuous source of MHC class II that can be therapeutically exploited [18].

Currently, no TCR-transgenic T cell therapy is evaluated in any clinical trial for glioma patients, while a total of 10 such trials are listed for peripheral solid tumors, such as advanced and metastatic malignancies which are specified only by antigen expression, incl. melanoma, non-small cell lung cancer, or triple negative breast cancer, all of which are located in the US (ClinicalTrials.gov.). Of these, four studies are recruiting, while three are active not-yet-recruiting studies and one has been terminated due to low accrual. Both completed trials come from the Jonsson Comprehensive Cancer Center, Los Angeles, CA, and used TCR-transgenic T cells directed against the tumor-associated cancer-testis antigens NY-ESO-1 and MART-1 in conjunction with antigen peptide-loaded dendritic cells. The phase 1 study additionally performed the adoptive cell transfer in combination with the checkpoint inhibitor Nivolumab to address safety and feasibility of this approach as well as persistence of administered T cells in solid cancers (NCT02775292). No results are currently publicly available for this trial. The preceding phase 2 study was restricted to melanoma patients and achieved initial tumor regression which lasted up to 6 months after start of study (NCT00910650)) [133]. From this trial, it was concluded that further improvements are needed to maintain and prolong responses.

#### Glioma-Specific Neoantigens for TCR T Cell Therapy

##### H3K27M

Diffuse midline gliomas harbor recurrent mutations in H3F3A and HIST1H3B (see the “Shared Neoantigen Targets” section). From a CD8+ T cell clone established by stimulation of HLA-A2+ CD8+ T cells with synthetic peptide encompassing the H3.3K27M mutation, complementary DNA for TCR α- and β-chains were cloned into a retroviral vector. TCR-transduced HLA-A2+ T cells efficiently killed HLA-A2+ H3.3K27M + glioma cells in an antigen- and HLA-specific manner. Adoptive transfer of TCR-transduced T cells significantly suppressed the progression of glioma xenografts in mice. These data provide a basis for developing T cell-based therapy targeting this shared neoepitope [134]. MultIceNTER Phase I Peptide VaCcine Trial for the Treatment of H3-Mutated Gliomas (INTERCEPT-H3) (NCT04808245) is an active not-yet-recruiting study applying atezolizumab and an H3K27M peptide vaccine that is administered in combination with topical Imiquimod. As the study exploits a long peptide targeting H3K27M, study treatment is not restricted to HLA-A2+ patients and enables the discovery of patient-individual TCRs binding H3K27M presented also on MHCII.

##### CICR215W

Up to 70% of oligodendrogliomas harbor recurrent mutations in the gene of capicua transcriptional repressor (CIC) [135]. One such example is a point mutation leading to the neoepitopic protein CICR215W. We have recently found that CICR215W harbors an MHC class II-restricted neoepitope that elicits specific and robust T helper cell responses in MHC-humanized A2.DR1 mice. Following establishment of CICR215W-specific T cell lines, we cloned and characterized a DR1-restricted murine TCR binding CICR215W but not CICwt. Intracerebroventricular T cell transfer of murine A2.DR1 T cells retrovirally overexpressing this DR1-restricted CICR215W-specific TCR in combination with low dose irradiation led to rejection of intracranial tumors in some mice [136]. This was the first observation that exploiting an MHC class II-restricted neoantigen-targeting TCR showed therapeutic efficacy when combined with low dose irradiation. Because of the moderate frequency of this specific neoepitope, TCR-transgenic T cell therapy targeting CICR215W could be further developed in patient-individual multivalent TCR-transgenic T cell approaches, but likely not as single therapeutic.

##### IDHR132H

In contrast to CIC mutations, IDH mutations are highly monomorphic and disease-defining in astrocytomas and oligodendrogliomas. Within the translational research program of the NOA-16 trial, an IDH1R132H-reactive T cell receptor was identified from a transcriptionally defined T cell clonotype from a pseudoprogressive lesion [47]. Whereas accessibility to post-vaccine relapse tissue was limited in NOA-16, peripheral and tumoral vaccine-induced T cell clonotypes will be systematically subjected to TCR discovery within the AMPLIFY-NEOVAC trial (NOA-21, NCT03893903). Especially vaccine trials with no HLA restriction as inclusion criterion, offer the opportunity of TCR warehouse generation. Further studies including HLA-alloreactivity assays using vaccine-induced and patient-individual TCRs are required to assess safety and feasibility of off-the shelf T helper cell neoepitope-specific TCRs targeting IDH1R132H.

#### Glioma-Associated Antigens for TCR T Cell Therapy

##### NLGN4X

As part of the premanufactured warehouse of 39 pre-defined unmutated HLA class I-presented antigens, a glioma-associated antigen processed and naturally presented from the NLGN4X protein, was safely targeted by the APVAC1 multimer peptide vaccine [11]. In a recent follow-up study, an NLGN4X-targeting HLA-A2-restricted TCR was lentivirally overexpressed in primary human T cells. Following intracerebroventricular transfer of NLGN4X-reactive TCR-transgenic T cells in NSG MHCI/MHC II knockout mice, experimental intracranial tumor growth was slowed down [137].

##### PTPRZ1

From one HLA-A2+ patient responding to APVAC1, autologous T cells reactive to protein tyrosine phosphatase receptor type zeta 1 (PTPRZ1) were sorted from PBMC and expanded in vitro. These PTPRZ1-reactive T cells exhibited dose-dependent cytotoxicity against peptide-loaded target cells and, more importantly, naturally PTPRZ1-expressing target cells in vitro [11]. These data validate not only the specific and functional reactivity of TCR-expressing T cells, but also the natural processing and presentation of these targets on HLA-A2+ tumor cells as a requirement for effective killing. PTPRZ1 belongs to the R5 subfamily of receptor-type protein tyrosine phosphatases (RPTP) [138]. PTPRZ1 has an extracellular carbonic anhydrase (CAH)-like domain and a fibronectin type III-like domain, and two intracellular tyrosine phosphatase domains [139]. Three isoforms are generated by alternative splicing from PTPRZ1: two transmembrane isoforms, PTPRZ-A and PTPRZ-B, and one secretory isoform, PTPRZ-S (also known as phosphacan); all isoforms are preferentially expressed in the CNS. Nevertheless, PTPRZ1 has been shown to be strongly overexpressed in malignant gliomas, especially glioblastoma [140, 141]. Interestingly, analyses of intratumoral heterogeneity revealed that the level of PTPRZ1 overexpression is strongly associated with cancer stemness, signified by the definition of PTPRZ1 transcripts as a stemness classifier gene [142]. While in an orthotopic xenograft model, an intrathecally administered cytotoxin saporin-conjugated antibody targeting PTPRZ1 delayed tumor growth of an intracranially injected human glioma cell line [143], following TCR discovery and validation, therapeutic efficacy of PTPRZ1-targeting TCRs is yet to be demonstrated.

Whereas TCR T cell therapy is still in its infancy, it holds great promise to complement CAR-exploiting approaches by multivalent cellular therapies or may present an alternative, when suitable surface antigens are scarce.

### Application Routes for Transgenic Cell Therapy

To date, 20 clinical trials are currently investigating or have investigated genetically modified cell therapies for brain tumor patients, all of which administer CAR T cells directed against a variety of mostly tumor-associated antigens. In many cases, chemotherapeutic lymphodepletion, mostly using cyclophosphamide and fludarabine, is administered prior to infusion. Cell therapy has shown remarkable clinical benefit in non-solid tumors and some solid tumor entities. For patients with GBM, T cell infiltration through the blood–brain barrier and potential on-target toxicity are of particular concern. To circumvent restricted homing of T cells to brain tumors after i.v. adoptive transfer, some clinical studies applying cellular therapy to glioma patients have already adapted to locoregional injections. In 15 cell therapy trials for brain tumor patients, cellular products are known to be administered locally, i.e., into the resection cavity, or intraventricularly via a reservoir. Evidence for superiority of locoregional administration over systemic infusion mainly comes from preclinical cell transfer experiments in — mostly immunodeficient — mouse brain tumor models.

While preclinical studies evaluating systemic CAR T cell therapy via i.v. injections have resulted in strong antitumor effects in peripheral solid tumor models, early preclinical T cell therapy attempts in intracranial tumor models demonstrated variable efficacy. T cell transfer experiments in an intracranial breast cancer model using either i.v. or intratumoral Her2-specific CAR T cell injections showed antitumor responses, whereas at the same time, i.v. injections of IL13Rα2-directed CAR T cells were reported to have no effect in a glioblastoma model [144, 145]. More recently, several preclinical studies reported enhanced antitumor efficacy of intraventricularly or locoregionally delivered CAR T cells directed against HER2, IL13α2, EPHA2, and B7-H3 compared to intraventricular delivery in several xenograft brain tumor models 125, 146, 147. Efficacy included increased survival and reduced systemic inflammatory cytokines levels. The conclusion from these and other studies that local administration of transgenic T cells is superior to systemic application, however, has to be interpreted with caution, because the effective trafficking of i.v. injected genetically manipulated T cells to the brain parenchyma requires cytokine gradients, which will not be established in immunodeficient mice. Similarly, tumor immune microenvironmental reprogramming and endogenous tumor-specific T cell responses will not occur in these mice, but have been demonstrated to be associated with clinical response to CAR T cell therapy in glioblastoma [148]. Especially for TCR-transgenic T cell therapy such as those targeting T helper cell epitopes, an MHC-proficient microenvironment of the host is a pre-requisite to evaluate therapeutic efficacy in pre-clinical brain tumor models. Effective migration of transferred T cells can be comprehensively studied in these immunocompetent mouse models.

Also, early clinical CAR T Cell trials used local delivery of the T cell product, such as IL13Rα2-specific CAR T cells injected directly into the resection cavity, eliciting minor adverse events [100]. Since then, other studies have applied CAR T cells intraventricularly, such as the actively recruiting IL13Rα2-directed CAR T cell trial NCT04661384, while a lot of studies compare different application routes, either by comparing several treatment arms or by consecutive change of the injection site, i.e., from i.v. to local infusion, according to study protocol. Another, not-yet-recruiting IL13Rα2 CAR T cell trial aims to compare several local types of infusion in different treatment arms, i.e., intraventricular versus the combination of intratumoral and intraventricular infusions versus infusions into the resection cavity (NCT02208362). Recent interim analysis of a phase I trial applying HER2-specific CAR T cells reported evidence for pseudoprogression and effective local induction of T cell-recruiting chemokines after locoregional delivery. The trial was composed of two arms comparing injections into the tumor cavity to intraventricular infusions (NCT03500991) [111]. Final results may shed light onto the question if one or the other administration route is superior. In the CAR2BRAIN phase I trial administering HER2-specific CAR NK cells, in the escalation cohort, cells are first administered intracranially into the wall of the resection cavity during relapse surgery (in patients with a planned partial or total resection) or into the tumor (in patients with a planned biopsy). In a planned expansion cohort, patients will additionally receive max. 12 weekly infusions through a Rickham reservoir, which will be implanted during relapse surgery (NCT03383978).

Some clinical evidence has recently been gained that locoregional administration of CAR T cells seems indeed to elicit more potent antitumor responses compared to i.v. injections. In the phase I dose escalation trial applying GD2-directed CAR T cells to patients with H3K27M mutant midline gliomas, eligible patients who exhibited clinical benefit from i.v. injections during the escalation phase, received intracerebroventricular infusions via an Ommaya reservoir, which was implanted during surgery (NCT04196413; see the “Glioma-Associated Antigens for CAR T Cell Therapy” section). Beneficial effects on local immune cell composition in the CSF were demonstrated to be most prominent at the peak inflammation time point after intracerebroventricular compared to i.v. infusions. These results indicate superiority of intracerebroventricular over i.v. administration regarding systemic toxicity, pro-inflammatory signaling circuits, and clinical improvement [117].

Further clinical studies which systematically compare systemic to different types of local injections, incl. those mentioned above, will support preclinical efforts to conclude on a cell therapy approach that is both most clinically effective in terms of antitumor response and safe in terms of minimizing adverse inflammatory events, in order to achieve maximal benefit for brain tumor patients.

## Technical Innovation and Outlook

Biomedical innovation has led to the development of the above-mentioned therapeutic concepts that are approaching or already have to prove themselves efficacious in the clinical arena. Importantly, technical innovation continuously proceeds, repetitively resulting in highly innovative approaches making it impossible to discuss them in their entirety. Therefore, we just name a few here: Tumor-specific neoepitopes may also result from frameshift mutations in tumor cells, the so-called frames. Frames represent potential immunogenic targets and can be utilized for tumor vaccination. A first clinical trial targeting frames has been conceptualized and will soon recruit patients with non-small cell lung cancer (NSCLC) to receive a multi-frame vaccine concomitant to the ICI Pembrolizumab (NCT04998474). Moreover, improvement in vector design of DNA vaccines but also DNA vectors to deliver CARs or TCRs to T cells is a field of tremendous technical innovation. Lopes et al. engineered vesicular stomatitis virus glycoprotein to be used as a carrier of foreign T cell tumor epitopes, which they termed plasmid to deliver T cell epitope (pTOP) [149], and Bozza et al. developed a nonviral, nonintegrating DNA nanovector platform for the safe, rapid, and persistent manufacture of recombinant T cells [150]. In the light of recent findings that CRISPR/Cas9-modified T cells frequently become aneuploidic, such episomal DNA vectors are of great therapeutic potential [151]. Single cell sequencing has enabled the high throughput discovery of patient-individual tumor-targeting TCRs by evaluation of their cognate cellular transcriptomes and tumor reactivity gene set definitions [152]. With the help of single cell sequencing, robotics, and molecular cloning, actively personalized TCR discovery from TIL is becoming clinically scalable. Also, novel optofluidic platforms may enable identification of tumor-reactivity even in an HLA- and target-agnostic way [153]. Ultimately, it will be of importance that such scalable platforms are GMP-compatible, and that providers of closed cell manufacturing and gene delivery devices systematically invest at the interfaces with each other but also with academic and comprehensive cancer centers that care for the patient. Summarizing only some of the current technical innovations here, it remains pure speculation which gene delivery technologies but also which vaccine targets will prevail in glioma immunotherapy. Undoubtedly, with the broadened accessibility to and applicability of cell therapies worldwide, cost-effective solutions such as DNA or RNA vectors will be advantageous. Regarding the optimal selection of vaccine targets, tumor-specificity, association with stemness, antigen persistence during disease progression, and strong epitope presentation are likely required for successful therapy development. All in all, the careful selection of patient-individual targets, immunotherapeutic modality, and application route remains to have a great potential to improve the survival of glioma patients.

## Supplementary Information

Below is the link to the electronic supplementary material.

Supplementary file1 (DOCX 34 kb)

Supplementary file2 (DOCX 34 kb)

Supplementary file3 (DOCX 32 kb)

Supplementary file4 (DOCX 34 kb)

Supplementary file5 (DOCX 34 kb)
